# Hæmorrhage after Parturition

**Published:** 1844-06-01

**Authors:** 


HÆMORRHAGE AFTER PARTURITION.

Dr. John Hall Davis narrates a case of flooding occurring both before and after the removal of the placenta, which was arrested at first by a large draught of cold water, and finally by the application of the T bandage and a compress. The patient did well. He concludes by observing, that in the management of this case, which required much personal exertion, he was entirely deprived of the services of the party previously in attendance, who, frightened at the serious aspect which the case put on, had taken to his heels before his arrival, and never returned.—*Ibid.*

